# Pathology and Molecular Diagnostics in the Personalized Treatment of Lung Diseases

**DOI:** 10.3390/jpm15120566

**Published:** 2025-11-24

**Authors:** Andrea Ambrosini-Spaltro

**Affiliations:** Pathology Unit, Morgagni-Pierantoni Hospital, AUSL Romagna, 47121 Forlì, Italy; andrea.ambrosinispaltro@auslromagna.it; Tel.: +39-0543-731703

## 1. Personalized Treatment in Both Neoplastic and Non-Neoplastic Lung Diseases

Personalized treatment specifically tailored to the patient’s needs is nowadays a successful and necessary therapeutic approach. Personalized treatment was initially introduced in lung carcinomas, which were among the first diseases to benefit from this new type of treatment and paradigm shift [1,2]. Each lung carcinoma needs to be analyzed, and specific modifications should be both identified and targeted to achieve a successful treatment. EGFR, KRAS, ALK, and ROS1 are some of the most well-known genes that can be targeted with specific drugs. Immunotherapy may also significantly contribute to this tailored approach using PD-L1 immunohistochemical expression [3]. Recent studies have also subdivided carcinoids into three different subgroups based on the different expression of three molecules (OTP, ASCL1, and HNF1A) with potential benefit from different therapeutic strategies [4]. This tailored treatment is important not only for neoplastic lung diseases, where it was traditionally and historically first introduced, but also for non-neoplastic lung diseases, where personalized treatments have been investigated only recently. Idiopathic pulmonary fibrosis (IPF) has been successfully treated with two different antifibrotic drugs, and a recent meta-analysis has supported the use of both antifibrotic treatments, underlining that they significantly reduce morbidity and mortality [5]. Some studies suggest that IPF can be further subtyped into different subgroups, each of which may respond differently to various modulators [6,7]. A personalized approach is currently under investigation in other non-neoplastic lung diseases, such as sarcoidosis [8].

## 2. Pathology in Personalized Treatment

As personalized treatments improve more and more, pathology and diagnostic procedures have gained considerable importance, as they are collectively responsible for both describing and analyzing the crucial predictive factors that drive the specific tailored treatments. An accurate diagnostic framework is of paramount importance, not only for identifying the correct disease but also for determining its subsequent therapy. In particular, pathology plays a pivotal role in this field, and its successful application relies on its two main components: morphology and molecular analysis.

There is a strict connection between a traditional morphological approach and novel molecular techniques. Both contribute to the diagnosis and allow each patient to obtain a specific, tailored therapy. Much of the literature of the last 10 years has highlighted the importance of a correct and comprehensive molecular profile to achieve accurate diagnosis and treatment, especially in lung tumors. However, this molecular profiling is as important as the traditional morphological assessment. First, the diagnosis starts with morphology, which is responsible for the correct initial diagnosis and subtyping of the lesion. Second, morphological examination guides the identification of the right tissue that needs to be molecularly analyzed: the most sophisticated molecular techniques cannot be correctly performed without accurate identification of neoplastic tissue and its different components. Together, these steps allow an adequate workflow, with correct tissue preservation, optimization, analysis, and finally, successful treatment for the patient.

## 3. Connections Between Neoplastic and Non-Neoplastic Lung Diseases

Non-neoplastic lung diseases, especially diffuse parenchymal lung diseases, are usually treated only by a restricted group of centers and specialists, whereas neoplastic lung diseases are usually managed more broadly. However, non-neoplastic lung diseases share many features with neoplastic lung diseases in terms of etiology, pathogenesis, diagnosis, and treatment. Many well-known risk factors for neoplastic lung disease are common with non-neoplastic diseases, such as smoking, pollution, and infections [9]. Morphological analysis of neoplastic diseases relies on a deep understanding of normal and non-neoplastic pulmonary pathology. For example, the invasiveness of lung adenocarcinomas is now much more clearly defined by the absence of normal elastic fibers, when elastic fibers are normally present in lung parenchyma [10]. Elastic fibers have also been well defined and analyzed in diffuse parenchymal lung disease [11]. Pathogenetically, it has been observed that IPF [12] and chronic obstructive pulmonary disease (COPD) [13] share common pathways with neoplastic diseases. Therefore, considering both non-neoplastic and neoplastic lung diseases together may help in better defining and understanding this anatomical district and achieving a more comprehensive knowledge of this complex field of study.

## 4. An Overview of Published Articles

This special issue is composed of six articles: four studies, one review, and one case report. Collectively, they cover a broad area of lung pathology, underlining the critical role of pathology in personalizing the treatment of lung diseases. This special issue has also been intentionally dedicated to both neoplastic and non-neoplastic disorders to reduce the gap between neoplastic and non-neoplastic lung diseases, which can be considered a continuum, a single unity with many different faces and mutual interconnections.

The review by Castelli et al. (contribution 1) investigated the relationship between interstitial lung disease and cancer. Interstitial lung diseases and lung carcinoma share several mechanisms and pathogenetic pathways. First, lung cancer incidence is higher in patients with interstitial lung diseases, probably because of the same risk factors and a common genetic background. The altered pathogenetic mechanisms that lead to fibrosis are also responsible for the development of carcinomas. The mutations most commonly shared by lung cancer and fibrosis are surfactant protein A (SFTPA) genes, telomere-related genes, and p53. In patients with lung cancer and interstitial lung diseases, traditional target mutations are rarely encountered, whereas BRAF and MET mutations are more frequently detected. The diagnostic and therapeutic approaches for lung cancer in interstitial lung diseases are diffusely and comprehensively described.

Lunardi et al. (contribution 2) also examined the relationship between non-neoplastic and neoplastic lung diseases by analyzing Chronic Obstructive Pulmonary Disease (COPD) and lung cancer. The authors examined morphology and molecular data from 110 lung adenocarcinomas in three groups of patients: 38 COPD smokers, 54 non-COPD smokers and 18 non-COPD non-smokers. Among the three groups, non-COPD non-smokers were significantly different from the other two groups, while COPD smokers and non-COPD smokers shared many morphological and molecular similarities. Non-COPD non-smokers showed a higher prevalence of lepidic pattern and a lower proliferative index. Conversely, both COPD smokers and non-COPD smokers shared many mutations (KRAS and PIK3CA) not found in non-COPD non-smokers, suggesting that smoking significantly and early impacts lung pathogenesis.

Barisione et al. (contribution 3) analyzed the role of Rapid On-Site Evaluation (ROSE) in interventional pneumology performed by pneumologists without the aid of a pathologist/cytopathologist. After examining 133 selected smears, they demonstrated that trained pulmonologists could correctly perform adequacy assessments. This is of particular importance in settings where pathologists/cytopathologists are not available or are located in separate places from pulmonologists in their routine practice.

Garlin Politis (contribution 4) analyzed the complex molecular signature of lung adenocarcinomas metastatic into the Central Nervous System. They collected 58 lung metastatic adenocarcinomas and subdivided them into early and late metastases. Collectively, 76% (44/58) of patients harbored driver mutations, with no significant difference between early and late metastases. The difference between early and late metastasis is only in survival, as early metastasis shows a worse outcome. Interestingly, the author also highlighted the importance of analyzing both DNA and RNA, as they collectively contribute to these important data.

Ambrosini-Spaltro et al. (contribution 5) described 14 cases of non-small cell lung carcinomas with NRAS mutations. They found that NRAS-mutated lung carcinomas may display sarcomatoid features, especially when examined in surgically resected specimens (in 2/5 cases, 40.0%). Furthermore, mutations in codon 61 were more frequent (9/13, 69.2%) and usually associated with a more favorable outcome than mutations involving other codons.

Centonza (contribution 6) reported a novel ALK translocation, the SPECC1L::ALK fusion variant. By molecular analysis, they demonstrated that the tyrosine kinase domain of the ALK protein was completely preserved. The patient was treated with three different target inhibitors: crizotinib, alectinib, and lorlatinib. This report highlights the importance of extensively analyzing the presence of specific translocations, even in common target alterations.

## 5. Dr. Giulio Rossi and His Memory

This special issue was originally conceived, structured, and planned by Dr. Giulio Rossi, an eminent Italian pulmonary pathologist, who unfortunately passed away during the creation of this Special Issue. His enthusiastic approach to pathology has inspired many of his colleagues. He always emphasized the importance of pathology in clinical practice and research. He also tried to help many young collaborators in their daily practice and routine activities.

I wanted to complete his project to honor his memory and celebrate his scientific value. His brilliant mind and overwhelming activity did not deserve to be abandoned in pursuit of such an important goal.

## 6. Conclusions

This Special Issue covers many different topics. Two main areas are (1) pathology as the initial and crucial step for both morphology and molecular analysis; and (2) mutual connections between neoplastic and non-neoplastic lung diseases. Research on personalized medicine could be addressed in the future especially in non-neoplastic lung diseases, following the example of the numerous studies performed on neoplastic lung diseases.
